# Supplementary material for: Entrainment of heterogeneous glycolytic oscillations in single cells
Source: Sci Rep. 2015 Mar 24;5:9404. doi: 10.1038/srep09404 (PMC4371117; doi:10.1038/srep09404)
Supplement: Supplementary Information [file srep09404-s1.pdf]

Entrainment of heterogeneous glycolytic oscillations in single cells

Anna-Karin Gustavsson, Caroline B. Adiels, Bernhard Mehlig, and Mattias Goksör*

Department of Physics, University of Gothenburg, SE-41296 Gothenburg, Sweden

*mattias.goksor@physics.gu.se

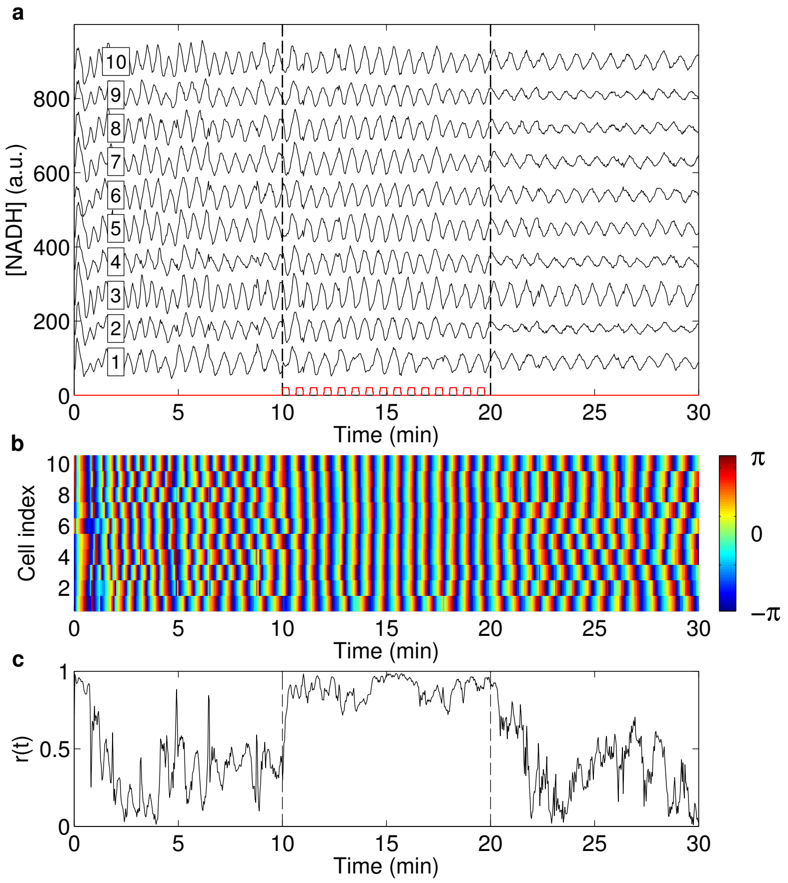

Supplementary Figure S1 Entrainment of oscillations by addition of acetaldehyde. (a) Graph showing the NADH fluorescence intensity from individual cells (cell index in box), where each trace is offset 90 units for clarity. Oscillations are induced at time 0 of the experiment by exposing the cells to a solution containing 20 mM glucose + 5 mM cyanide. After 10 min, entrainment by acetaldehyde (ACA) addition is investigated by periodically changing the flow rates in the inlet channels of the microfluidic flow chamber, causing a periodic change between the glucose/cyanide solution and a glucose/cyanide solution with 1 mM ACA with a period time of 40 s for a total of 15 periods. The time intervals during which the cells are exposed to ACA are marked in red. As can be seen, the amplitude of the oscillations remains relatively unaffected by the perturbation. **(b)** Graph showing the phases of the oscillations of the individual cells shown in **(a)**. Here it can be seen that the phases are shifted as the cells become entrained by the perturbation. **(c)** Graph showing the time dependent order parameter, calculated from the phases shown in **(b)**. An order parameter close to unity indicates synchronisation, while a low value indicates a large heterogeneity of the phases. Here it is thus clear that the cells become entrained by the periodic addition of ACA through phase shifts.

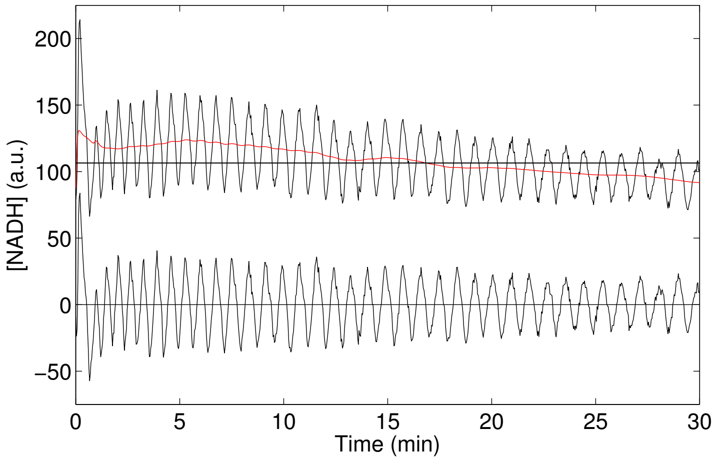

Supplementary Figure S2 Running average correction of NADH signal required for analysis of phases. Graph showing the NADH fluorescence intensity from a single oscillating cell (top), where the straight black line shows the total average of the signal and the red line shows the running average calculated with a window of approximately two periods. Below, the same signal after subtracting the running average is shown. After subtracting the running average, the signal oscillates around 0, which is a requirement for extraction of the instantaneous phase of the signal.
